# Menstrual fluid-derived small extracellular vesicles: a novel reservoir with distinct molecular signatures and implications for endometriosis etiopathology

**DOI:** 10.1093/hropen/hoag020

**Published:** 2026-03-15

**Authors:** Vicente Peragallo-Papic, Paz Cerda-Castro, Aliosha I Figueroa-Valdés, Hugo E Tobar, Patricia Valdebenito, Manuel B Donoso, Benjamín Bustos, Javiera Yakcich, Reyna Peñailillo, Gino Nardocci, Stephanie Acuña-Gallardo, Sebastián E Illanes, Francisca Alcayaga-Miranda, Lara J Monteiro

**Affiliations:** Program in Biology of Reproduction, Centre for Biomedical Research and Innovation (CiiB), Universidad de los Andes, Santiago, Chile; PhD Program in Biomedicine, Faculty of Medicine, Universidad de los Andes, Santiago, Chile; Program in Biology of Reproduction, Centre for Biomedical Research and Innovation (CiiB), Universidad de los Andes, Santiago, Chile; IMPACT, Center of Interventional Medicine for Precision and Advanced Cellular Therapy, Universidad de los Andes, Santiago, Chile; IMPACT, Center of Interventional Medicine for Precision and Advanced Cellular Therapy, Universidad de los Andes, Santiago, Chile; IMPACT, Center of Interventional Medicine for Precision and Advanced Cellular Therapy, Universidad de los Andes, Santiago, Chile; Program in Biology of Reproduction, Centre for Biomedical Research and Innovation (CiiB), Universidad de los Andes, Santiago, Chile; IMPACT, Center of Interventional Medicine for Precision and Advanced Cellular Therapy, Universidad de los Andes, Santiago, Chile; Faculty of Medicine, Universidad de los Andes, Santiago, Chile; Obstetrics and Gynecology Unit, Clínica Universidad de los Andes, Santiago, Chile; Faculty of Medicine, Universidad de los Andes, Santiago, Chile; Obstetrics and Gynecology Unit, Clínica Universidad de los Andes, Santiago, Chile; Faculty of Medicine, Universidad de los Andes, Santiago, Chile; Obstetrics and Gynecology Unit, Clínica Universidad de los Andes, Santiago, Chile; Program in Biology of Reproduction, Centre for Biomedical Research and Innovation (CiiB), Universidad de los Andes, Santiago, Chile; IMPACT, Center of Interventional Medicine for Precision and Advanced Cellular Therapy, Universidad de los Andes, Santiago, Chile; IMPACT, Center of Interventional Medicine for Precision and Advanced Cellular Therapy, Universidad de los Andes, Santiago, Chile; Faculty of Medicine, Universidad de los Andes, Santiago, Chile; Molecular Biology and Bioinformatics Lab, Program in Molecular Biology and Bioinformatics, Centre for Biomedical Research and Innovation (CiiB), Universidad de los Andes, Santiago, Chile; Program in Biology of Reproduction, Centre for Biomedical Research and Innovation (CiiB), Universidad de los Andes, Santiago, Chile; IMPACT, Center of Interventional Medicine for Precision and Advanced Cellular Therapy, Universidad de los Andes, Santiago, Chile; Program in Biology of Reproduction, Centre for Biomedical Research and Innovation (CiiB), Universidad de los Andes, Santiago, Chile; IMPACT, Center of Interventional Medicine for Precision and Advanced Cellular Therapy, Universidad de los Andes, Santiago, Chile; Faculty of Medicine, Universidad de los Andes, Santiago, Chile; Obstetrics and Gynecology Unit, Clínica Universidad de los Andes, Santiago, Chile; Department of Obstetrics & Gynaecology, Yong Loo Lin School of Medicine, National University of Singapore, Singapore, Singapore; IMPACT, Center of Interventional Medicine for Precision and Advanced Cellular Therapy, Universidad de los Andes, Santiago, Chile; Faculty of Medicine, Universidad de los Andes, Santiago, Chile; Laboratory of Nano-Regenerative Medicine, Centre for Biomedical Research and Innovation (CiiB), Universidad de los Andes, Santiago, Chile; Program in Biology of Reproduction, Centre for Biomedical Research and Innovation (CiiB), Universidad de los Andes, Santiago, Chile; IMPACT, Center of Interventional Medicine for Precision and Advanced Cellular Therapy, Universidad de los Andes, Santiago, Chile; Faculty of Medicine, Universidad de los Andes, Santiago, Chile

**Keywords:** menstrual fluid, small extracellular vesicles, endometriosis, transcriptomics, non-invasive biomarkers, sEVs biomarkers

## Abstract

**STUDY QUESTION:**

Can the transcriptomic profile of small extracellular vesicles (sEVs) derived from menstrual fluid (MF) provide preliminary insights into their potential roles as biomarkers and mediators in endometriosis (EM) progression?

**SUMMARY ANSWER:**

MF-derived sEVs from EM patients display altered molecular signatures and enhanced proangiogenic activity *in vitro*, suggesting their potential involvement in EM pathophysiology.

**WHAT IS KNOWN ALREADY:**

EM is a chronic gynecological disorder affecting ∼10% of reproductive-age women, associated with pain, infertility, and delayed diagnosis. sEVs carry cargo that resembles their cellular origin, making them promising biomarkers for various diseases. While sEVs have previously been isolated from MF and their protein content has been analyzed, their transcriptomic content and biological function remain unexplored.

**STUDY DESIGN, SIZE, DURATION:**

Two cross-sectional studies were conducted. The first compared MF-sEVs between nulliparous (n = 10) and multiparous (n = 12) women. The second compared MF-sEVs from EM patients (n = 10) and Controls (n = 11). Participants were recruited between 2021 and 2023 from a tertiary care center in Santiago, Chile.

**PARTICIPANTS/MATERIALS, SETTING, METHODS:**

MF from healthy (nulliparous and multiparous) and EM patients was self-collected using a menstrual cup on Day 2 of menstruation, and peripheral blood of a subset of healthy women was obtained by venipuncture. sEVs were isolated from the plasma fraction of MF and from peripheral blood plasma. Nanoparticle tracking analysis, transmission electron microscopy, and flow cytometry were used to assess sEVs concentration, size, morphology, and tetraspanin expression. Next-generation sequencing was performed to analyze MF-derived sEVs' transcriptomic profiles, and endothelial tubulogenesis assays using human umbilical vein endothelial cells (HUVECs) assessed angiogenic function *in vitro*.

**MAIN RESULTS AND THE ROLE OF CHANCE:**

MF-derived sEVs of healthy women were significantly more abundant than those isolated from their peripheral blood (*P *= 0.008), and they exhibited typical morphology and tetraspanin expression. Neither parity nor disease affected MF-sEVs' concentration or size; however, CD63 expression was significantly reduced in sEVs from nulliparous women and EM patients (*P *= 0.02). Transcriptomic analysis revealed unique mRNAs and long noncoding RNAs (lncRNAs) signatures that reflect their potential contribution to disease etiopathology. Functionally, MF-derived sEVs from EM patients (MF-EM-derived sEVs), significantly enhanced tubule formation *in vitro* (*P *< 0.05), indicating proangiogenic potential in EM.

**LARGE SCALE DATA:**

The raw sequencing data have been deposited in the NCBI GEO database under the accession number GSE310627.

**LIMITATIONS, REASONS FOR CAUTION:**

The sample size limits the generalizability of findings. The RNA-seq component should be interpreted as a discovery-phase analysis; while it reveals potential molecular differences in MF-derived sEVs from women with EM, these results are preliminary and require validation in larger, independent cohorts. Functional validation was restricted to *in vitro* assays; *in vivo* studies are needed to confirm biological effects and mechanisms underlying the MF-EM-derived sEVs proangiogenic effects.

**WIDER IMPLICATIONS OF THE FINDINGS:**

The non-invasive isolation and disease-associated transcriptomic signatures of MF-sEVs-enriched isolates highlight their potential as biomarkers for EM although validation is required in an independent and larger cohort to assess their robustness and clinical utility. Currently, EM diagnosis relies on invasive laparoscopic surgery, often resulting in diagnostic delays. The proangiogenic effect of MF-sEVs observed *in vitro* suggests that their cargo may reflect disease progression. These findings are in line with a recent report that described the proteome of MF-sEVs from EM patients, demonstrating their involvement in mesothelial barrier disruption, which reinforces the role of MF-sEVs in EM etiopathology.

**STUDY FUNDING/COMPETING INTEREST(S):**

This work was supported by the National Agency for Investigation and Development-ANID, FONDECYT Regular No. 1230932, No. 1230875, No. 1241103, FONDECYT POSTDOCTORADO No. 3230201, FAIN-UANDES No. 202201, and ANID-Basal funding for Scientific and Technological Center of Excellence, IMPACT No. FB210024. The authors declare no conflicts of interest.

WHAT DOES THIS MEAN FOR PATIENTS? Endometriosis is a condition that affects millions of women and can cause pain, fatigue, and infertility. Unfortunately, getting a proper diagnosis often takes 6–9 years. This is because the tests currently available are not always accurate, and confirming the diagnosis usually requires surgery. As a result, many women wait until symptoms become very severe before receiving care. This study explored a new, less invasive way to help diagnose endometriosis, by analyzing menstrual fluid, i.e. the blood and tissue released during the menstrual period. Menstrual fluid is easy to collect and may carry important information about what is happening inside the uterus.

Our research focused on tiny particles found in menstrual fluid, called small extracellular vesicles. These particles are released by cells and carry signals reflecting the state of the lining of the uterus. When we compared these particles from women with and without endometriosis, we found that their contents were different. The particles from women with endometriosis carried signals linked to the disease and showed the ability to promote the formation of new blood vessels, a process involved in the progression of endometriosis.

These findings suggest that menstrual fluid might one day be used to help diagnose endometriosis earlier and more easily. This could lead to faster treatment and better care for women living with the condition.

## Introduction

Small extracellular vesicles (sEVs) are heterogeneous, nano-scale cell-derived structures enclosed by a lipid bilayer, that have been broadly classified into ectosomes and exosomes based on their biogenesis (Théry *et al.*, 2018; Kalluri and LeBleu, 2020; van de Wakker *et al.*, 2024). sEVs are characterized by the presence of the surface tetraspanins CD9, CD63, and CD81, and the internal cytosolic markers syntenin-1, caveolins, flotillins-1 and 2, and cytoskeletal proteins (Théry *et al.*, 2018; Jankovicova *et al.*, 2020). They have been extensively studied in the past few decades because of their important role in biological processes, cell–cell communication, their involvement in the pathogenesis of various diseases, and as potential source of biomarkers (Berumen Sanchez *et al.*, 2021). These functions are proposed to be mediated by the contents of sEVs, which reflects their cell of origin as well as its functional state. The content of sEVs includes nucleic acids, lipids, metabolites, and cytosolic and cell-surface proteins (Théry *et al.*, 2018; Mathieu *et al.*, 2019; Kalluri and LeBleu, 2020; Berumen Sanchez *et al.*, 2021; Ciferri *et al.*, 2021). sEVs have been reported to offer a window into altered cellular or tissue states, and their detection in biological fluids potentially offers a multicomponent diagnostic readout (Kalluri and LeBleu, 2020; van de Wakker *et al.*, 2024). Indeed, sEVs have been reported in a plethora of biological fluids and in regard to various diseases (Mathieu *et al.*, 2019; Berumen Sanchez *et al.*, 2021; Ciferri *et al.*, 2021), including several gynecological disorders (Rai *et al.*, 2021; Brennan *et al.*, 2025; Gurung *et al.*, 2025).

Endometriosis (EM) is a chronic, debilitating disease that affects around 10% of women of reproductive age worldwide, and 30–50% of these women struggle with infertility (Hufnagel *et al.*, 2015; Horne and Missmer, 2022). It is characterized by the presence of endometrial-like tissue outside the uterus, causing chronic pelvic pain, dysmenorrhea, dyspareunia, dysuria, dyschezia, irregular uterine bleeding, and fatigue (Sarria-Santamera *et al.*, 2020; Taylor *et al.*, 2021). Due to the multivariate range of symptoms that it can present and the inexistence of a biomarker, EM is under-diagnosed and associated with a 6.7-year mean latency from onset of symptoms to definitive diagnosis (Husby *et al.*, 2003; Zondervan *et al.*, 2018; De Corte *et al.*, 2025). Although the cellular and molecular origins of EM remain a subject of debate, the most widely accepted hypothesis is Sampson’s theory of retrograde menstruation, which postulates that menstrual fluid (MF) travels backward through the Fallopian tubes and deposits outside the uterine cavity, leading to the development of ectopic endometrial lesions (Sampson, 1927; Halme *et al.*, 1984; Bulletti *et al.*, 2002). While retrograde menstruation occurs during a low-estrogen phase of the cycle, the subsequent implantation and progression of ectopic lesions are strongly influenced by rising estrogen levels and an estrogen-rich microenvironment. Estrogen promotes the survival, migration, and invasion of ectopic endometrial cells and facilitates angiogenesis, especially when coupled with an impaired immune response that fails to eliminate refluxed tissue (Chantalat *et al.*, 2020; Saunders and Horne, 2021; Horne and Missmer, 2022). This evidence highlights MF as a biologically relevant and accessible source for studying the etiopathogenesis of EM and for identifying potential biomarkers through noninvasive approaches (Wong *et al.*, 2018; Brennan *et al.*, 2025; Dosnon *et al.*, 2025). While laparoscopic visualization followed by histological confirmation remains the gold standard for diagnosis and staging, its invasive nature restricts its use to patients with advanced disease or to those who fail medical management, thus resulting in significant diagnostic delays (Wong *et al.*, 2018; Zondervan *et al.*, 2018, 2020; Brennan *et al.*, 2025; Dosnon *et al.*, 2025). Consequently, the development of alternative, minimally invasive diagnostic tools is a pressing clinical need. In this context, MF represents a promising substrate for biomarker discovery and mechanistic studies, as it can be collected safely and repeatedly using noninvasive procedures and reflects the endometrial and uterine microenvironment (Tindal *et al.*, 2024). Its complex cellular and molecular composition, including blood cells, immune and stem/progenitor cells, endometrial fragments, cervicovaginal secretions, and secreted factors such as proteins and extracellular vesicles, further supports its use as a rich biological matrix for exploring disease-associated processes (Tindal *et al.*, 2024; Gurung *et al.*, 2025).

A growing body of evidence suggests that sEVs play an important role in the pathogenesis of EM by mediating intercellular communication (Gurung *et al.*, 2025). sEVs derived from endometriotic lesions and other body fluids, such as plasma, peritoneal fluid, and tubal fluid, carry distinctive cargoes that influence key disease processes, including endometriotic cell proliferation, immune evasion, angiogenesis, and invasion (Khalaj *et al.*, 2019; Nazri *et al.*, 2020, 2023; Chu *et al.*, 2024; Gurung *et al.*, 2025). Despite extensive research on sEVs in EM, studies on MF-derived sEVs (MF-sEVs) remain scarce. While proteomic analyses have been conducted, their transcriptomic landscape remains unexplored (Gurung *et al.*, 2025). Given the accessibility of MF and its direct relevance to the origins of EM, our study aims to fill in this gap by characterizing sEVs isolated from MF of healthy women and those with EM. This approach provides critical insights into their role in disease pathogenesis and paves the way for identifying novel biomarkers for the noninvasive diagnosis of EM.

## Materials and methods

### Patient enrollment

These two cross-sectional studies included women of reproductive age (18–45 years old) attending the Obstetrics and Gynecology Unit of Clínica Universidad de los Andes, Santiago, Chile. The first case-study included nulliparous (women who have never delivered a viable infant) or multiparous (women who have delivered one or more babies) women, and the second case-study included women without EM (controls), confirmed either by surgical visualization (during a previous cesarean section) or by transvaginal ultrasound with bowel preparation, and women with laparoscopic confirmation of stage III–IV EM (scored at surgery according to the (American Association of Gynecologic Laparoscopists) classification; Abrao *et al.*, 2021). Absence or presence of EM was reported by clinicians. Participants who had not used contraceptive steroids, gonadotropin-releasing hormone agonists, or progestins for at least 3 months and who had provided written informed consent were recruited to donate MF and peripheral blood. The study protocols were reviewed and approved by the Ethical Scientific Committee of Universidad de los Andes (CEC202025, date of approval 22 April 2020; CEC2022107, date of approval 24 October 2022). The demographic and gynecological characteristics of participants, including age, weight, height, age of menarche, parity, pregnancy loss, and pain were recorded at the time of MF collection. Pain intensity was recorded using a visual analogue scale (VAS, 0–10). Women with clinical signs of menopause, colorectal and/or urinary tract pathology, or a history of any gynecological condition, including adenomyosis, uterine fibroids, ovarian cysts, endometrial polyps, or uterine malformations, were excluded from this study.

### Isolation of sEVs from the plasma of MF and peripheral blood

MF was self-collected overnight using a hypoallergenic silicone menstrual cup within the first 48 h of menstruation onset. Samples were then transferred into a 50 ml tube containing transport media [10 ml PBS 1× (Cytiva, South Logan, UT, USA), 0.25 mg/ml amphotericin B (Biological Industries, Beit Hamek, Israel), 1% penicillin/streptomycin (P/S) (Life Technologies, Grand Island, NY, USA), 0.3% sodium citrate (Sigma Aldrich, St. Louis, MO, USA)], and immediately transported to the laboratory at 4 °C to be processed within 4 h of sample collection (Varas-Godoy *et al.*, 2019; Penailillo *et al.*, 2022). A minimum of 3 ml of MF was required for sEVs isolation. Peripheral blood was collected in two BD Vacutainer citrate tubes (0.109M, 3.2% sodium citrate) (BD Biosciences, San Jose, CA, USA) whenever it was possible for the patients to visit the study midwives during the same menstrual cycle in which MF was obtained. Plasma from peripheral blood and MF was obtained using Ficoll-Paque Plus (GE Healthcare, Amersham, UK; 1.077 g/ml) density gradient by centrifugation at 400 × *g* for 30 min at room temperature (RT) (without acceleration or brake). For sEVs isolation, plasma samples obtained from MF and peripheral blood were subjected to serial centrifugation (without acceleration or brake) at 300 × *g* for 10 min, 2000 × *g* for 20 min, 10 000 × *g* for 40 min, and 110 000 × *g* for 60 min. The resulting pellet was resuspended in 10 ml PBS 1×, filtered through a 0.22 µm acetate–nitrocellulose membrane (EDLAB, Chang’an, China), and ultracentrifuged at 110 000 × *g* for 60 min. The pellet was then washed with 10 ml of PBS 1× and subjected to a final ultracentrifugation at 110 000 × *g* for 60 min. The sEVs-enriched pellet was recovered and stored at −80°C until further analysis. All centrifugation steps were performed at 4 °C.

### Nanoparticle tracking analysis

Particle concentration and size distribution were assessed using Nanoparticle Tracking Analysis (NTA) on a NanoSight NS300system (Malvern Instruments Limited, Worcestershire, UK) (Zavala *et al.*, 2020; Figueroa-Valdes *et al.*, 2021). In brief, five videos of 60 s each were recorded using a camera level of 8, slider gains of 15, and analyzed with a detection threshold of 3. Particle concentration was normalized to the volume of MF from which it was isolated.

### Flow cytometry analysis

MF sEVs' surface markers were assessed by flow cytometry using a FACS CANTO II flow cytometer (BD Biosciences), following methodology described previously (Figueroa-Valdes *et al.*, 2021; Figueroa-Valdés *et al.*, 2025). Briefly, 1.4 × 10^9^ particles were resuspended in PBS 1× (Cytiva) and incubated with aldehyde/sulfate latex beads (Thermo Fisher Scientific, Rockford, IL, USA) for 16 h at 4 °C. Samples were then blocked with 0.33M glycine solution (United States Biological, Salem, MA, USA) for 1 h at RT. Beads were centrifuged at 8000 × *g* for 2 min at 4 °C, resuspended in 10% w/v bovine serum albumin (BSA) (Winkler Ltda., Santiago, Chile) in PBS 1×, and incubated for 45 min at RT. The pellet was divided into four tubes and incubated in 2% BSA containing either 0.025 mg/ml of the primary antibodies CD9 (clone M-L13), CD63 (clone H5C6), CD81 (clone JS-81), or 5 μl of the isotype control (clone X40) (all from BD Biosciences) for 30 min at RT. After washing with PBS 1×, samples were blocked with 10% BSA for 30 min at RT. The pellet was incubated in 2% BSA with Alexa Fluor 488-conjugated anti-mouse secondary antibody (clone RMG1-1) (BioLegend, San Diego, CA, USA) (final concentration 0.025 mg/ml) for 30 min at RT. Finally, samples were washed three times with PBS 1×, and the pellet was resuspended in PBS 1× for acquisition on the flow cytometer. All incubations were performed with agitation in PBS 1×.

For data analysis, the bead population was gated, and doublets were excluded. Within this population, the fold change of the median fluorescence intensity (MFI) was determined relative to the isotype control. MFI was used as a quantitative measure since bead-based assays do not allow discrimination of individual populations. Data analysis was performed using FlowJo software (v10.9.0; Ashland, OR, USA).

### Western-blot analysis

Western-blot analysis was performed using 32 μl of sEVs samples, which were run on a 4–20% gradient acrylamide gel. Primary antibodies against Syntenin-1 (Novus Biologicals, Centennial, CO, USA) and Apolipoprotein A1 (Apo A-I) were used, followed by detection with a horseradish peroxidase-linked anti-rabbit secondary antibody (LI-COR, Lincoln, NE, USA). Protein bands were visualized using the Pierce ECL Western Blotting Substrate detection system (Thermo Fisher Scientific). Signal acquisition was performed using the iBright CL 1500 Imaging System (Thermo Fisher Scientific). Protein quantification was normalized to the total protein content of each sample (Supplementary Fig. S1) using the No-Stain Protein Labeling Reagent (Invitrogen), and normalization factors were calculated with the iBright Analysis Software (Thermo Fisher Scientific). In line with MISEV2023 recommendations, intracellular compartment markers are considered optional for extracellular vesicle preparations derived from cell-free biofluids such as MF plasma, and therefore, these were not assessed (Welsh *et al.*, 2024).

### Transmission electron microscopy

sEVs morphology and integrity were visualized using transmission electron microscopy (TEM) as described previously (Zavala *et al.*, 2020; Figueroa-Valdes *et al.*, 2021). Briefly, 1.95 × 10^9^ particles were deposited onto formvar/carbon-coated copper meshes (Electron Microscopy Sciences, Hatfield, PA, USA) for 1 min, followed by negative staining with 15 μl of 2% (w/v) uranyl acetate solution for 1 min and dried at 37 °C for 15 min. Imaging was performed at the Advanced Microscopy Facility UMA (Pontificia Universidad Católica de Chile, Santiago, Chile) using a TALOS TEM (Thermo Fisher Scientific). Representative images were captured at 57 000× magnification.

### RNA isolation and RNA sequencing

RNA was isolated from MF plasma-derived sEVs using TRIzol LS (Invitrogen, USA) on a 60 µl sEVs sample volume. RNA concentration was assessed with a NanoDrop One (Thermo Fisher Scientific). A minimum of 250 ng RNA was used for RNA sequencing (RNA-Seq). RNA-Seq was performed by Azenta Life Sciences (South Plainfield, NJ, USA). Briefly, library preparation was performed with rRNA depletion, and sequencing was carried out on an Illumina next-generation sequencing platform with paired-end 150 bp reads. DESeq2 (Love *et al.*, 2014) normalized counts were used for downstream analyses. Genes with a log2(Fold Change) ≥ 0.5 and a −log_10_(*P* value) ≥ 1.301 were considered differentially enriched in sEVs. Data analysis was conducted using R (v4.2.3) (R Core Team, 2023), and graphs and heatmaps were generated using the ggplot2 (v3.4.4) (Wickham, 2016) package. Gene Ontology (GO) enrichment analysis was performed using the clusterProfiler (v4.6.2) (Yu *et al.*, 2012) package.

### Angiogenesis assay

Immortalized human umbilical vein endothelial cells (HUVEC) were maintained in Dulbecco’s Modified Eagle Medium (DMEM) supplemented with 1% penicillin/streptomycin, 1% amphotericin, and 10% fetal bovine serum (FBS). For the angiogenesis experiments, 70 µl of ice-cold growth factor-reduced phenol-red-free MATRIGEL matrix (Corning Life Sciences, Union City, CA, USA) was added to each experimental well of a 96-well plate and incubated at 37 °C for 30 min to allow polymerization. Each MF-sEV sample was tested in triplicate. HUVECs (1 × 10^4^ cells/well) were seeded in either endothelial growth medium (EGM) (Bullet Kit, Lonza, Verviers, Belgium) as a positive control or in DMEM supplemented with 1% penicillin/streptomycin, 1% amphotericin, and 2% FBS. Except for the positive control, each well was exposed to either PBS 1× (vehicle control) or 1.5 × 10^4^ particles/cell and incubated for 4 h at 37 °C in 5% CO_2_ to allow tubule formation. Subsequently, tubule formation was examined using an inverted microscope (Primo Vert, Zeiss, Jena, Germany), and one image per well was acquired at 4× magnification using an AxioCam ERc5s camera (Zeiss), capturing the entire surface of the well to avoid subjective field selection. Quantification of nodes, junctions, meshes, segments, and branches was performed with the ImageJ-Angiogenesis Analyzer-HUVEC Phase Contrast software v1.0.c. Complete 4× images were loaded to the ImageJ software, and the Angiogenesis Analyzer plug-in was applied using the ‘Analyze HUVEC Phase Contrast’ setting. This fully automated process quantified the number of nodes, junctions, meshes, segments, and branches, which were subsequently recorded for statistical analysis (Supplementary Fig. S2). Although image acquisition was performed using a standardized protocol to avoid field selection bias, blinding was not applied. As quantification was fully automated, the risk of measurement bias is minimal, but this remains a minor study limitation.

### Statistical analysis

Graphing and statistical analyses were performed using GraphPad Prism 9.0 (GraphPad Software, La Jolla, CA, USA, v 9.2.0). Results were expressed as the mean ± SEM. Data normality was tested by the Shapiro−Wilk test and for analysis of two groups, *t*-test or Mann−Whitney *U*-test was used. Results were deemed statistically significant if the *P* value ≤ 0.05.

## Results

### Characterization of MF-derived sEVs from nulliparous and multiparous women

sEVs have been widely reported as promising non-invasive biomarkers for the diagnosis and prognosis of various diseases, including cancer, and neurological and gynecological disorders (Asleh *et al.*, 2023; Lee *et al.*, 2023; Bhavsar *et al.*, 2024; Zanirati *et al.*, 2024). Likewise, MF has gained attention as a biological reservoir of disease biomarkers, especially for gynecologic and obstetric diseases (Malik *et al.*, 2006; da Silva *et al.*, 2014; Tortorella *et al.*, 2014). Here we have successfully isolated sEVs from the plasma fraction of MF, ensuring efficient separation from potential non-sEVs contaminants.

**Figure 1.**
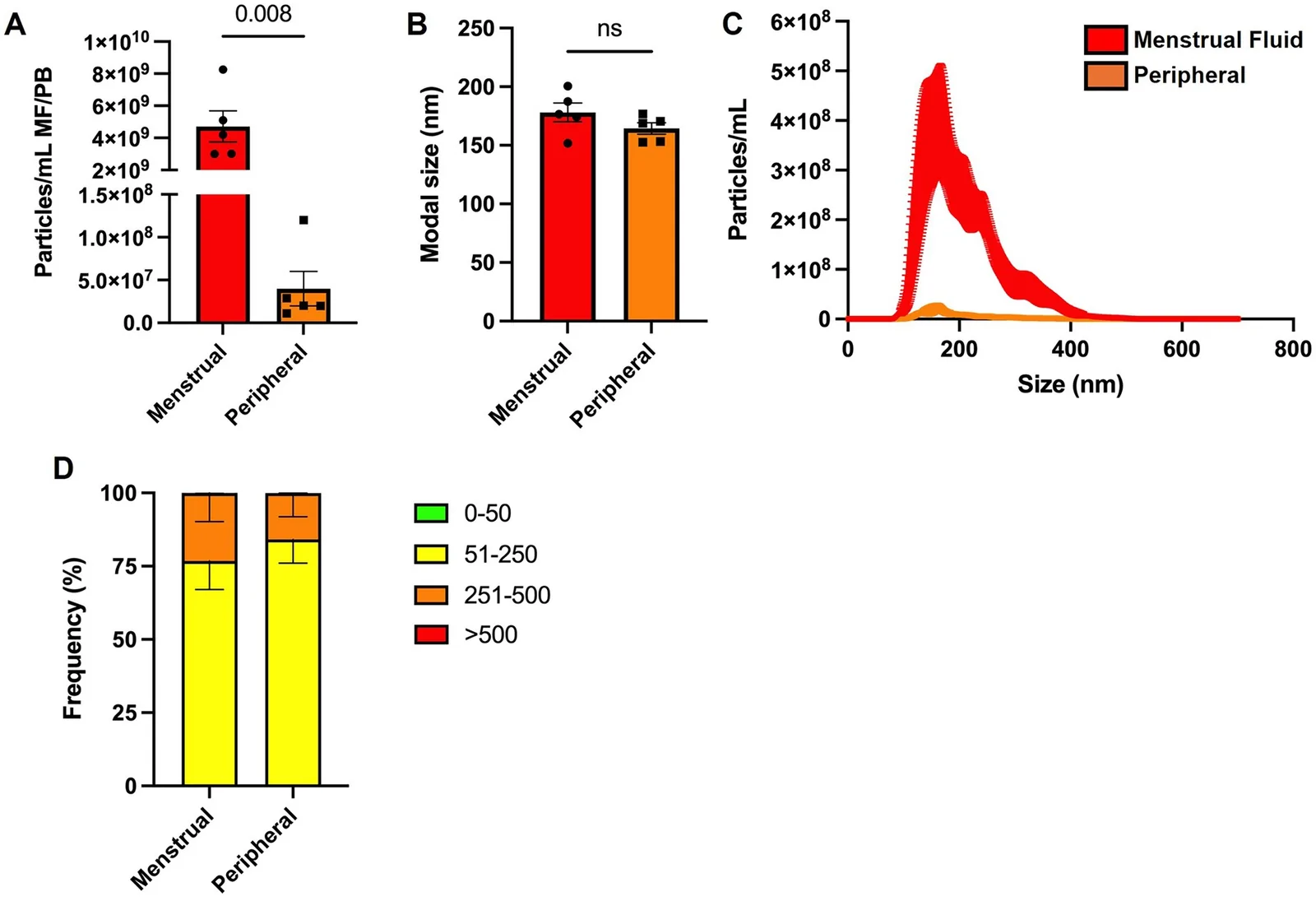
**Menstrual fluid serves as a good source of small extracellular vesicles (sEVs) compared to peripheral blood.** sEVs were isolated from the plasma fraction of peripheral blood and menstrual fluid collected from the same healthy donors (n = 5). (**A**) Particle concentration, (**B**) modal diameter, (**C**) size distribution, and (**D**) percentage size frequency determined by nanoparticle tracking analysis (NTA). Comparisons between groups were performed using the Mann–Whitney test. The data are represented as mean ± SEM. ns, non-significant; MF, menstrual fluid; PB, peripheral blood.

Our initial objective was to assess whether MF serves as a good source of biomarkers compared to a traditional biofluid, such as peripheral blood plasma. To this end, we isolated sEVs from the plasma of both peripheral blood and MF obtained from the same donors, analyzing equivalent blood volumes. Quantification by NTA revealed a significantly higher concentration of particles in MF compared to peripheral blood (*P *= 0.008) (Fig. 1A). While peripheral blood has been traditionally considered a key source for extracellular vesicle studies, these findings indicate that MF may represent a more abundant reservoir of sEVs. Beyond concentration, size analysis showed that MF-particles exhibit comparable modal sizes to those obtained from peripheral blood, and a positive skewness curve of size distribution, with most particles falling within the expected 50–200 nm range (Fig. 1B–D). The size distribution profile revealed a peak between 150 and 200 nm, unveiling the potential enrichment of sEVs. However, a small fraction of larger particles (251–500 nm) was also detected (Fig. 1D). These findings indicate that MF-particles share biophysical properties with peripheral blood-derived particles while also containing a minor proportion of larger particles.

**Figure 2.**
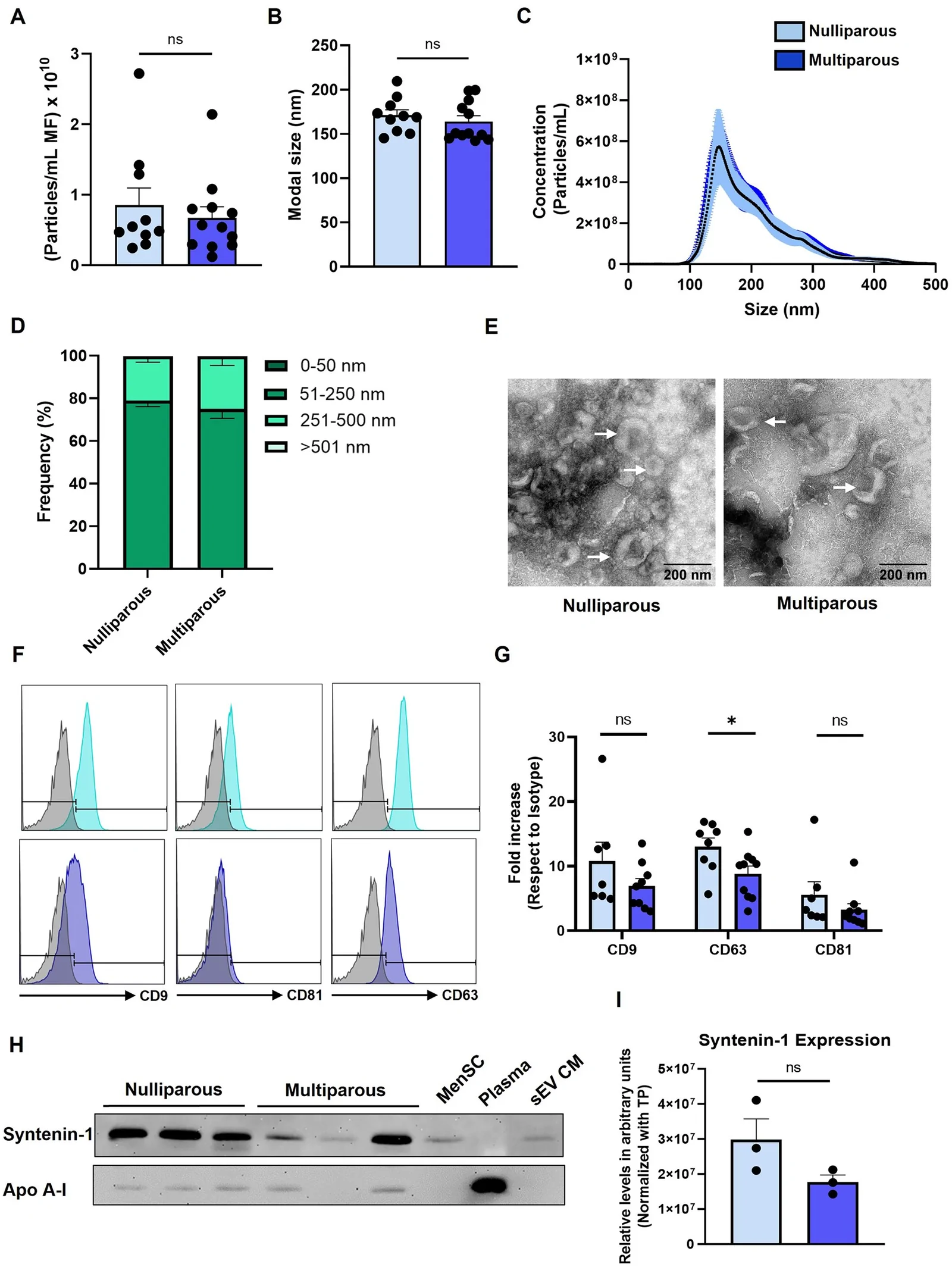
**Characteristics of menstrual fluid-derived small extracellular vesicles (MF-sEVs) from nulliparous and multiparous women.** sEVs were isolated from the plasma fraction of menstrual fluid from nulliparous (n = 10) and multiparous (n = 12) donors using a protocol combining serial filtration and centrifugation. (**A**) Particle concentration normalized by menstrual fluid (MF) volume, (**B**) modal diameter, (**C**) size distribution, and (**D**) percentage size frequency were determined by nanoparticle tracking analysis (NTA). (**E**) Representative transmission electron microscopy (TEM) images of MF-sEVs at magnification 57 000×. (**F**) Representative histograms depict tetraspanin profiles of sEVs analyzed by flow cytometry. Gray represents the isotype negative control, light blue represents nulliparous, and blue represents multiparous donors. (**G**) Graph represents the fold increase of CD9, CD63, and CD81 proteins relative to the isotype control. (**H**) sEVs were lysed and analyzed by western blot to assess the protein expression of Syntenin-1 and Apolipoprotein A-I (Apo A-I) (representative image of three nulliparous and three multiparous donors). sEVs derived from the conditioning media (sEVs CM) of menstrual mesenchymal stem cells (MenSCs) and MenSCs lysates were used as a positive control for the presence of syntenin-1. The supernatant obtained after the first ultracentrifugation of MF-derived plasma was included as a negative control for the presence of sEVs. (**I**) Synthetin-1 protein expression was normalized to the total protein content of each sample using the iBright CL 1500 Imaging System. Graphs present mean ± SEM. Statistical analysis was performed using the Mann–Whitney test. *, *P* ≤ 0.05, significant; ns, non-significant. R.F.U., relative fluorescence units.

We next sought to establish the identity of these particles as sEVs, and to determine whether parity status influences their characteristics. This comparison aimed to assess whether sEVs profiles are consistent among healthy women with different reproductive histories. Establishing such consistency is essential for postulating sEVs as potential biomarkers for EM, as it supports their stability and reproducibility across physiological variations. For this purpose, we compared MF-particles from nulliparous and multiparous women to assess potential differences in concentration, size distribution, and morphology. The demographic and clinical characteristics of both groups are summarized in Table 1. As expected, multiparous women had a significantly higher age and had experienced more gestations and birth deliveries compared to nulliparous women. NTA quantification revealed that the concentration of MF-particles was comparable between nulliparous (8.54 × 10^9^ particles/ml) and multiparous (6.72 × 10^9^ particles/ml) women, with no significant differences between groups (Fig. 2A). Similarly, the modal size of the particles was consistent across both groups, with average values of 171.1 nm for nulliparous and 164.0 nm for multiparous women (Fig. 2B). These findings suggest that the parity status does not substantially alter the overall abundance or basal size properties of MF-particles. To further assess the size distribution of particles within each group, we analyzed the proportion of particles falling within specific nanometric ranges. As shown in Fig. 2C, both nulliparous and multiparous MF-particles exhibited a similar distribution profile with positive skewness curves, with most vesicles falling within the expected 50–200 nm range, reinforcing their potential classification as sEVs. However, when analyzing the frequency of particle sizes (Fig. 2D), we also observed that a small fraction of particles exceeded 250 nm, particularly in the multiparous group. This suggests the potential presence of larger EVs, such as ectosomes or apoptotic bodies, which typically range from 200 nm to 1 µm. This subtle shift in size distribution may reflect physiological differences in vesicle subpopulations or biogenesis mechanisms between these groups. Finally, to confirm the structure, integrity, and morphology of the isolated particles, TEM was carried out on MF particles. Representative micrographs (Fig. 2E) revealed the presence of cup-shaped vesicles in both groups, a characteristic feature of sEVs. This morphological consistency further supports the fact that regardless of parity status, MF-particles maintain a well-defined ultrastructure. To further confirm the identity of these sEVs-enriched isolates, MISEV2023 guidelines were followed (Welsh *et al.*, 2024). Bead-based flow cytometry was performed to assess the presence of the well-characterized tetraspanins CD9, CD63, and CD81. As shown in Fig. 2F, MF-particles from both nulliparous and multiparous women expressed all three markers, supporting their classification as sEVs. From this point forward, we will refer to these vesicles as MF-sEVs, acknowledging that these represent sEV-enriched isolates in accordance with MISEV2023 guidelines (Welsh *et al.*, 2024). Quantitative comparison of MFI by flow cytometry (Fig. 2G) revealed that CD63 expression was significantly higher in sEVs from nulliparous women compared to those from multiparous women, whereas CD9 and CD81 levels remained similar between groups. Since CD63 is predominantly associated with endosomal-derived exosomes, this result suggests that sEVs from nulliparous women may contain a higher proportion of exosomes, while those from multiparous women could have a greater fraction of vesicles from alternative biogenesis pathways. To further explore the molecular composition of these vesicles, we assessed the expression of Syntenin-1, a cytosolic protein enriched in exosomes due to its role in ESCRT-dependent vesicle formation (Kugeratski *et al.*, 2021). Western-blot analysis (Fig. 2H) revealed that Syntenin-1 levels showed a tendency to be higher in sEVs from nulliparous women, although this difference did not reach statistical significance (Fig. 2I). This observation aligns with the increased CD63 expression, suggesting that sEVs from nulliparous women may be relatively enriched in exosomal subpopulations compared to those from multiparous women. Since MF contains a complex mixture of cellular and molecular components, we next evaluated the purity of the isolated sEVs by detecting Apo A-I, a well-established non-vesicular extracellular particle (NVEP) marker associated with plasma-derived sEVs (Karimi *et al.*, 2018). As shown in Fig. 2H, Apo A-I was strongly detected in the plasma control, whereas only faint bands were observed in MF-sEVs preparations. This pattern indicates a marked depletion, although not complete, of Apo A-I-containing lipoprotein particles by our isolation protocol. Intracellular compartment markers were not assessed, as these are considered optional under MISEV2023 guidelines, particularly for isolates derived from cell-free biofluids such as MF plasma, where the risk of cellular contamination is inherently low (Welsh *et al.*, 2024).

**Table 1.** Demographic and clinical characteristics of nulliparous and multiparous women.

Characteristics	Nulliparous (*n *= 10)	Multiparous (*n *= 12)	*P*-value
Age (years)	30.2 ± 6.9	38.3 ± 4.6	0.004
Weight (kg)	60.4 ± 7.7	59.7 ± 9.2	0.85
Height (m)	1.6 ± 0.07	1.63 ± 0.05	0.44
BMI (kg/m^2^)	23.2 ± 2.6	22.4 ± 3.3	0.53
Gestation Number	0.1 ± 0.3	3.2 ± 1.3	<0.0001***
Parity Number	0 ± 0.0	2.9 ± 0.8	<0.0001***
Pregnancy loss	0.1 ± 0.3	0.3 ± 0.6	0.50

Data are presented by n of mean ± SD. Statistical analysis was performed using the Student’s *t*-test.

*** *P* ≤ 0.001, significant.

Taken together, these findings suggest that while MF-sEVs from both nulliparous and multiparous women share common molecular markers but subtle differences in CD63 expression, and a trend toward increased Syntenin-1 levels in nulliparous-derived sEVs indicate that parity may influence the relative proportions of exosomal versus non-exosomal vesicles. These variations in sEVs composition highlight the potential for MF-sEVs to reflect physiological differences between individuals, prompting us to explore whether their characteristics were also altered in pathological conditions such as EM.

### Characterization of MF-derived sEVs from control and EM patients

EM is a gynecological disorder characterized by the ectopic implantation of endometrial-like tissue, with retrograde menstruation proposed as a key mechanism in its pathogenesis (Sampson’s theory). Since sEVs mediate cell–cell communication and have been implicated in various pathophysiological processes, we sought to characterize and compare MF-sEVs from healthy women and patients with EM to identify potential disease-associated alterations. The demographic and clinical characteristics of both groups are summarized in Table 2. As expected, symptoms typically associated with EM, such as dysmenorrhea and dyschezia, were significantly more frequent among patients with EM. The persistence of pain-related symptoms among patients with EM is consistent with the recurrent and multifactorial nature of the disease and aligns with previous reports describing limited long-term efficacy of current therapies (Zondervan *et al.*, 2018). However, because information regarding medication use was not collected in this cohort, it remains unclear whether these symptoms were influenced by ongoing pharmacological treatment or by the intrinsic recurrence of the disease.

**Table 2.** Demographic and clinical characteristics of healthy women and women with endometriosis.

Characteristics	Control (*n *= 11)	Endometriosis (*n *= 10)	*P*-value
Age (years)	34.0 ± 5.3	35.7 ± 3.9	0.41
Weight (kg)	60.5 ± 8.5	60.5 ± 5.0	>0.99
Height (m)	1.61 ± 0.06	1.62 ± 0.05	0.77
BMI (kg/m^2^)	23.3 ± 4.5	23.2 ± 2.8	0.96
Menarche (years)	13.0 ± 1.5	11.7 ± 1.0	0.04
Gestation Number	1.6 ± 1.8	1.1 ± 1.3	0.45
Parity Number	1.3 ± 1.4	0.7 ± 1.1	0.30
Pregnancy loss	0.4 ± 0.7	0.4 ± 0.7	0.90
Dysmenorrhea (0–10)	3.6 ± 1.7	7.8 ± 1.6	<0.0001***
Dyspareunia (0–10)	0.7 ± 1.1	3.1 ± 2.7	0.02
Dysuria (0–10)	0	1.2 ± 3.2	0.22
Dyschezia (0–10)	1.2 ± 1.8	5.9 ± 2.4	<0.0001***

Pain is rated from 0 to 10 using the visual analytical scale. Dysmenorrhea, non-cyclical or non-menstrual pelvic pain. Dyspareunia, intracoital pain. Dysuria, painful urination. Dyschezia, pain when defecating. Data are presented by n of mean ± SD. Statistical analysis was performed using the Student’s *t*-test.

*** *P* ≤ 0.001, significant.

**Figure 3.**
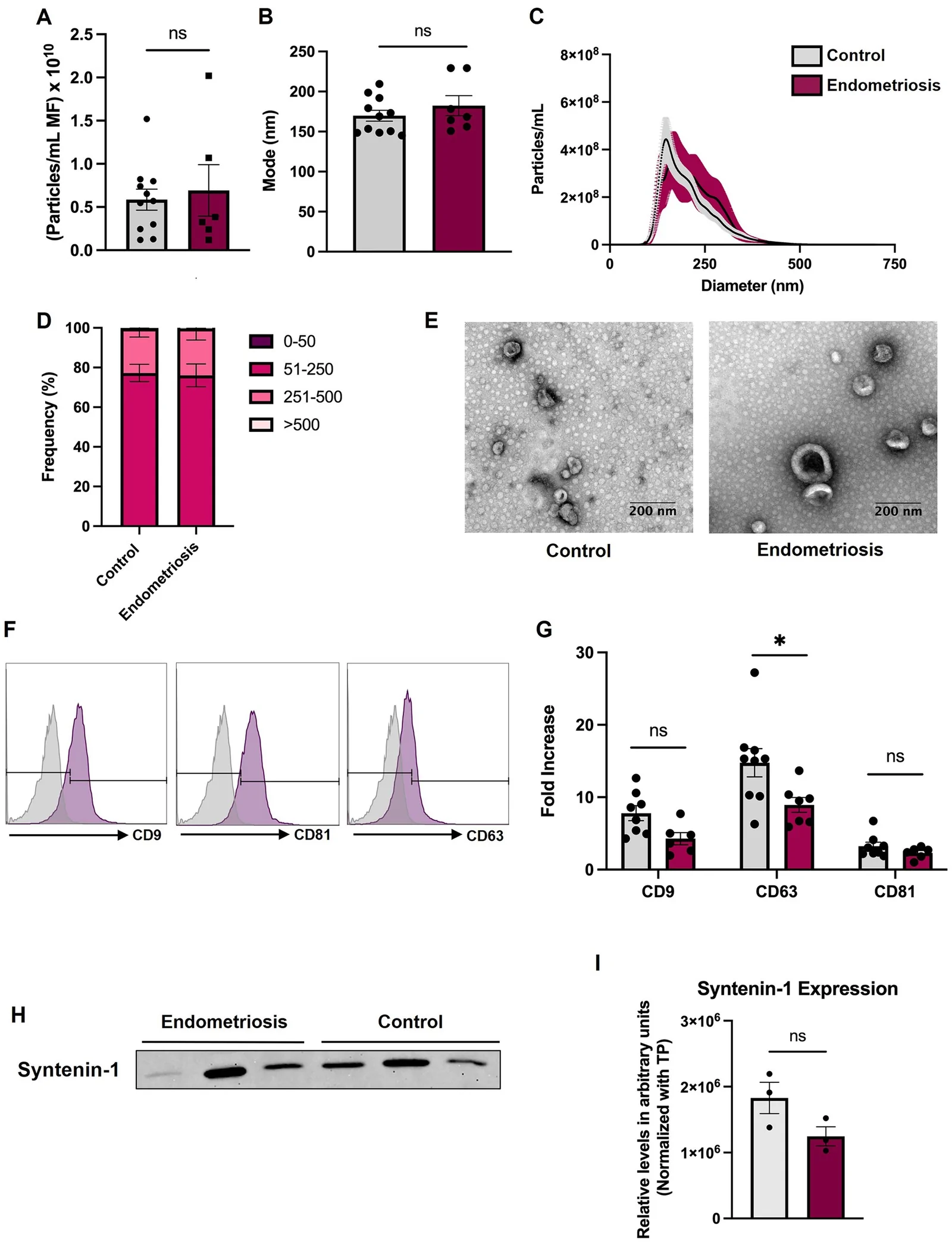
**Characteristics of menstrual fluid-derived small extracellular vesicles (MF-sEVs) from control and endometriosis women.** sEVs were isolated from the plasma fraction of menstrual fluid from controls (n = 11) and endometriosis (n = 7) donors using a protocol combining serial filtration and centrifugation. (**A**) Particle concentration normalized by MF volume, (**B**) modal diameter, (**C**) size distribution, and (**D**) percentage size frequency were determined by nanoparticle tracking analysis (NTA). (**E**) Representative transmission electron microscopy (TEM) images of MF-sEVs at magnification 57 000×. (**F**) Representative histograms depict tetraspanin profiles of sEVs analyzed by flow cytometry. Gray represents the isotype negative control, and deep red represents endometriosis women. (**G**) Graph represents the fold increase of CD9, CD63, and CD81 proteins relative to the isotype control. (**H**) sEVs were lysed and analyzed by western blotting to assess Syntenin-1 protein expression (representative image of three endometriosis and three controls donors). (**I**) Synthetin-1 protein expression was normalized to the total protein content of each sample using the iBright CL 1500 Imaging System. Graphs present mean ± SEM. Statistical analysis was performed using the Mann–Whitney test. *, *P* ≤ 0.05; ns, non-significant. R.F.U., relative fluorescence units.

To determine whether EM influences MF-sEVs concentration or size, we performed NTA quantification. As shown in Fig. 3A and B, no significant differences were found in particle concentration or modal size between control and MF-derived sEVs from EM patients (MF-EM-derived sEVs). This suggests that, despite the altered inflammatory and hormonal environment existent in the context of EM, the total yield and baseline size characteristics of MF-sEVs remain largely unaltered. We next examined the size distribution of vesicles to identify potential shifts in sEVs subpopulations. As depicted in Fig. 3C, while most sEVs from both groups fell within the expected size range and showed unimodal profiles with positive skewness curves, EM-derived sEVs exhibited an increased proportion of larger particles compared to controls. However, when analyzing the relative abundance of these larger vesicles, no statistically significant differences were observed (Fig. 3D). These findings suggest that while MF-EM-derived sEVs may contain a greater fraction of larger sEVs, this effect does not result in a drastic shift in the overall sEVs size profile. To further confirm the morphology and integrity of isolated MF-sEVs, we performed TEM imaging. As shown in Fig. 3E, cup-shaped vesicles were observed in both control and MF-EM-derived samples, reinforcing that the isolated particles correspond to bona fide sEVs regardless of disease status.

To investigate whether EM alters sEVs molecular composition, we assessed the presence of CD9, CD63, and CD81 by bead-based flow cytometry. As shown in Fig. 3F, sEVs from both control and EM samples exhibited detectable levels of all three tetraspanins. However, when quantifying MFI (Fig. 3G), we observed a significant reduction in CD63 expression in MF-EM-derived sEVs compared to controls, while CD9 and CD81 also exhibited a downward trend, though without reaching statistical significance. Since CD63 is closely associated with endosomal-origin exosomes, its reduced expression in MF-EM-derived sEVs suggests a potential shift in vesicle biogenesis pathways or alterations in the proportion of exosomal versus non-exosomal sEVs in EM. To further examine the endosomal origin of these vesicles, we assessed the expression of Syntenin-1 by western-blot analysis. As shown in Fig. 3H, Syntenin-1 was detected in all tested samples, confirming that at least a fraction of both MF control- and EM-derived sEVs originated from the endosomal pathway, with a slight, non-significant enrichment in control samples compared to MF-EM-derived sEVs (Fig. 3I).

Overall, these results indicate that while MF-sEVs from healthy and EM patients exhibit comparable concentration, size, and morphology, EM may alter sEVs subpopulations, particularly through a reduction in CD63 expression, along with a downward trend in CD9, CD81, and Syntenin-1 levels. This suggests that MF-EM-derived sEVs may differ functionally from their healthy counterparts.

### MF-derived sEVs from EM patients hold proangiogenic molecular signature and induce tubule formation *in vitro*

**Figure 4.**
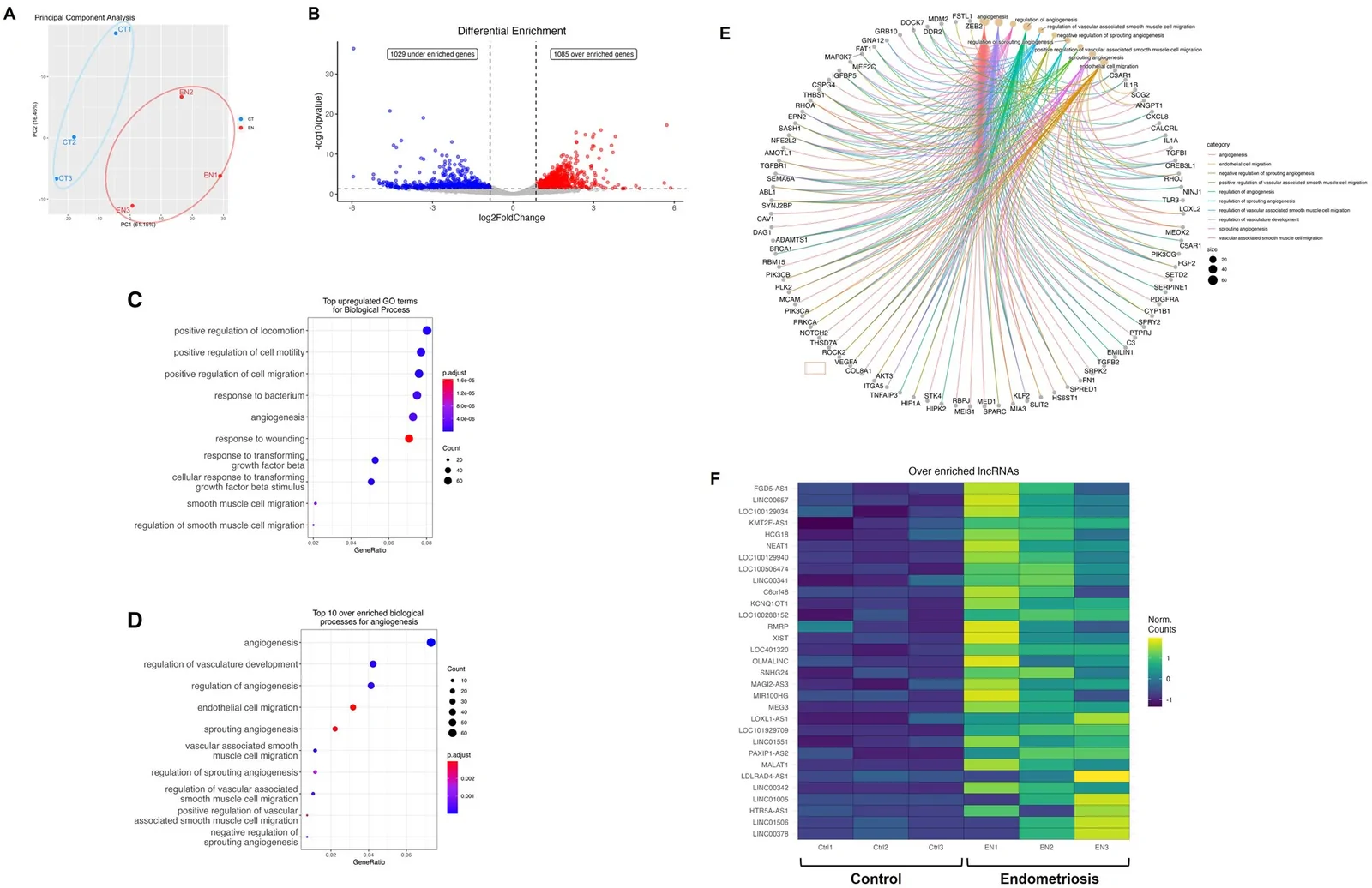
**Transcriptomic analysis of menstrual fluid-derived small extracellular vesicles (MF-sEVs) reveals unique mRNA and lncRNA signatures associated with endometriosis.** (**A**) Principal component analysis (PCA) of MF-sEVs from control (n = 3) and endometriosis (n = 3) donors show demarcations between groups. (**B**) Volcano plot representing 1085 overenriched genes (red) and 1029 underenriched genes (blue) in endometriosis MF-sEVs compared to controls MF-sEVs. (**C**) The top 10 significantly over-represented biological processes in endometriosis MF-sEVs, identified through Gene Ontology (GO) enrichment analysis. (**D**) The top 10 overrepresented biological processes related to the term ‘angiogenesis’ in endometriosis MF-sEVs, as determined by GO enrichment analysis. (**E**) Chord diagram illustrates the relationship between overenriched genes in MF-sEVs from endometriosis patients and their involvement in distinct GO angiogenesis-related processes. Each colored line represents a connection between a specific gene and a biological process. (**F**) Heatmap of overenriched long noncoding (lnc) RNAs in endometriosis MF-sEVs compared to controls MF-sEVs.

Having established that MF-sEVs from EM patients exhibit alterations in tetraspanin expression, we next sought to investigate their RNA cargo and functional implications. Since sEVs can transfer bioactive molecules, including mRNAs and non-coding RNAs, to recipient cells, we performed RNA-sequencing (RNA-Seq) analysis to preliminarily determine whether MF-EM-derived sEVs carry a distinct transcriptomic signature. As shown in Fig. 4A, principal component analysis (PCA) demonstrated that MF-sEVs from EM patients and healthy controls formed distinct transcriptional clusters, indicating significant differences in their RNA composition. Differential gene enrichment analysis revealed a total of 1029 under-enriched and 1085 over-enriched genes in MF-EM-derived sEVs compared to healthy controls (Fig. 4B, Supplementary Table S1), suggesting substantial transcriptomic alterations associated with the disease state. To determine the biological relevance of these transcriptomic differences, we performed GO enrichment analysis. Among the top 10 over-enriched biological processes in EM, we identified cell motility, migration, angiogenesis, and response to transforming growth factor beta (TGF-β) (Fig. 4C), all of which are critical in lesion establishment and EM progression. Several factors previously characterized as either up- or down-regulated in EM, including *MME* (CD10) (Atiya *et al.*, 2022), *CCN1* (IGFBP10) (Absenger *et al.*, 2004), *LIF* (Zutautas *et al.*, 2023), and *PGR* (Zhang and Wang, 2023) were identified within this dataset, strongly supporting the EM origin of sEVs and reflecting the importance of MF-EM-derived sEVs cargo as a representative snapshot of the molecular dynamics occurring in EM (Supplementary Fig. S3A). These results serve as a first step toward validating MF-EM-sEVs as biomarkers for EM and should be interpreted as discovery-phase insights. Validation in larger cohorts should be performed to determine their clinical readiness.

Given the well-established role of angiogenesis in EM pathogenesis, we further examined angiogenesis-related gene enrichment (Fig. 4D) and identified a statistically significant overrepresentation of angiogenesis-associated biological processes in MF-EM-derived sEVs. Several key proangiogenic factors, including *IL1B*, *CXCL8*, *TGFBI*, *FGF2*, and *VEGFA* were found to be highly enriched in EM-derived sEVs (Fig. 4E, Supplementary Fig. S3B), reinforcing their potential role in the vascular remodeling characteristics of endometriotic lesions. Additionally, among the 31 overenriched lncRNAs identified in MF-EM-derived sEVs, *NEAT1* (Zhou *et al.*, 2019; Liu *et al.*, 2023; Meng *et al.*, 2023), *XIST* (Hu *et al.*, 2019), *KCNQ1OT1* (Li *et al.*, 2022), *MEG3* (Ruan *et al.*, 2018), and *MALAT1* (Fig. 4F) (Huang *et al.*, 2018; Hou *et al.*, 2020; Vimalraj *et al.*, 2021) have been reported to be involved in molecular pathways that promote angiogenesis. These findings suggest that MF-sEVs from EM patients not only contain distinct RNA signatures but also carry an enriched pool of proangiogenic transcripts, raising the possibility that they actively contribute to the vascularization of ectopic endometrial lesions. To assess whether these transcriptomic differences translated into functional effects, we evaluated the capacity of MF-EM-derived sEVs to promote angiogenesis *in vitro*.

**Figure 5.**
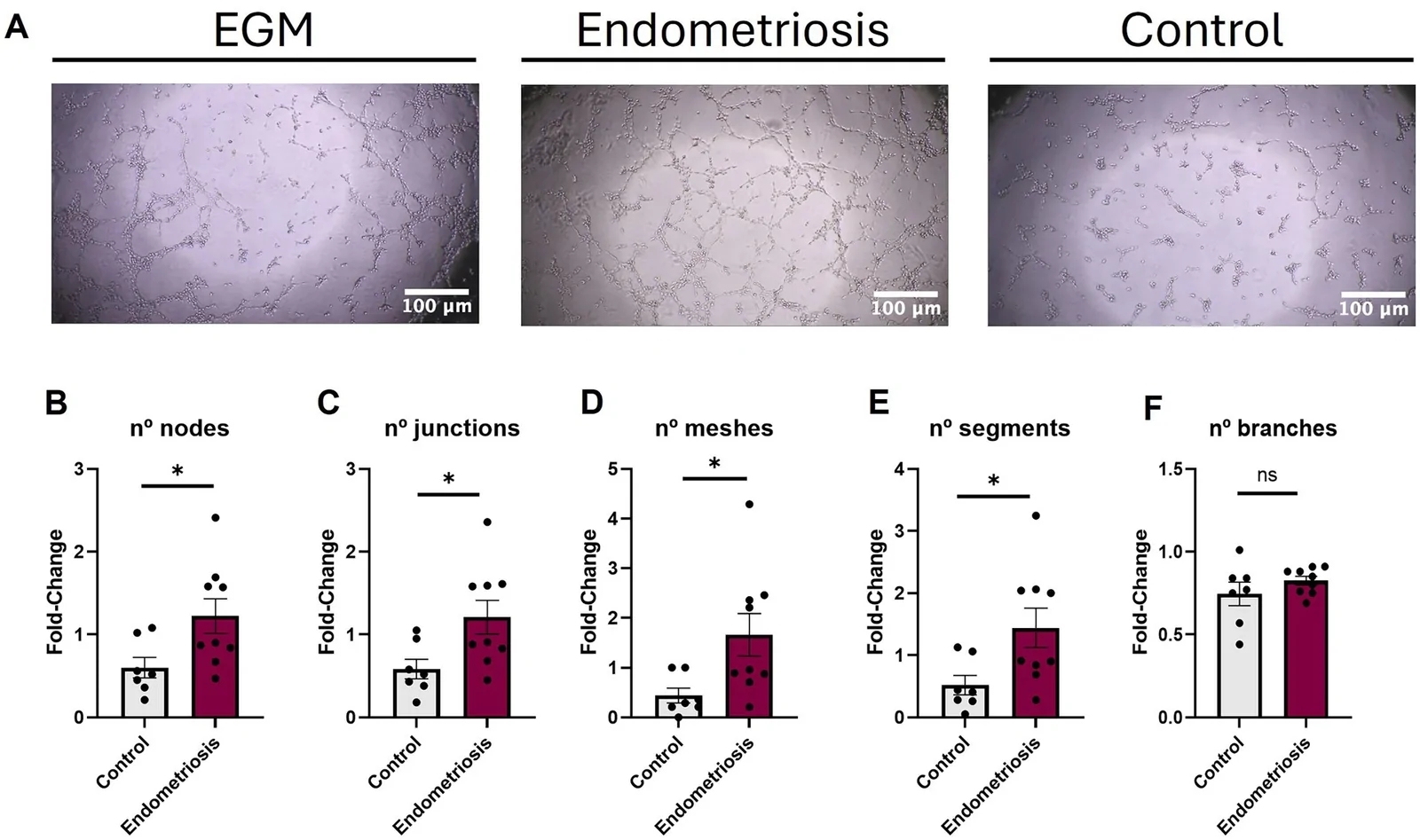
**Menstrual fluid-derived small extracellular vesicles (MF-sEVs) from women with endometriosis promote tubule formation in human umbilical vein endothelial cells (HUVECs).** Immortalized Human umbilical vein endothelial cells (HUVECs) were incubated with MF-sEVs (1.5 × 10^4^ particles/cell) from control (n = 11) and endometriosis (n = 10) patients. Endothelial growth medium (EGM) was used as positive control. (**A**) Representative images show tubule formation after 4 h (left panel). All images were captured at 4× magnification, covering the entire well surface. Each MF-sEV sample was tested in triplicate. (**B**) Graphs represent the quantitative analysis of B. nodes, (**C**) meshes, (**D**) junctions, (**E**) segments, and (**F**) branches. Data are presented as mean ± SEM. Statistical analysis was performed using the Mann–Whitney test. *, *P* ≤ 0.05; ns, non-significant.

We conducted a tubule formation assay using HUVECs to assess whether exposure to these particles enhanced angiogenic potential. Representative images of the tubule formation assay are shown in Fig. 5A, illustrating the structural differences in endothelial network formation across experimental conditions. As depicted in Fig. 5B–F, HUVECs treated with MF-sEVs from EM patients exhibited a significant increase in key tubule formation parameters, including nodes, junctions, meshes, and segments, compared to cells exposed to MF-sEVs from healthy donors. Overall, MF-EM-derived sEVs exhibited a pronounced proangiogenic effect. Given the critical role of angiogenesis in EM progression, these findings support the idea that MF-EM-derived sEVs actively contribute to the vascular remodeling characteristic of the disease.

## Discussion

MF has long been overlooked as a biological source for EV research, despite its dynamic cellular composition and potential to reflect physiological and pathological states. In this study, we have successfully isolated and characterized sEVs from the plasma fraction of MF, confirming its use as a novel and abundant source of these vesicles. Compared to peripheral blood, MF-derived sEVs exhibited a significantly higher particle concentration while maintaining similar size distributions and canonical sEVs markers, highlighting its suitability for extracellular vesicle studies. The presence of a minor fraction of larger vesicles suggests that MF may also contain additional extracellular vesicle subtypes, reflecting the complex cellular environment of the endometrium. The low-level detection of Apo A-I in MF-sEVs preparations should be interpreted considering the emerging concept of an extracellular vesicle biomolecular corona. Recent work has demonstrated that abundant plasma proteins, including Apo A-I, can form a stable protein corona on the EV surface and remain associated after conventional purification steps, such as ultracentrifugation and density-gradient separation (Tóth *et al.*, 2021; Buzas, 2022). Thus, the presence of Apo A-I in MF-sEVs fractions likely reflects a small subset of corona proteins rather than substantial lipoprotein contamination.

Pregnancy and childbirth are known to induce lasting alterations in the structure and biology of the endometrium (Gamliel *et al.*, 2018; Shi *et al.*, 2023), yet its influence on the composition of MF-derived sEVs had not been explored. Our findings indicate that while parity does not significantly affect sEVs concentration or modal size, nulliparous-derived sEVs exhibited higher CD63 expression and a trend toward increased Syntenin-1 levels, suggesting an enrichment of exosomal subpopulations. This shift in vesicle composition could reflect parity-induced adaptations in endometrial regeneration, cellular turnover, or vesicle biogenesis pathways.

The impact of these findings became more pronounced when considered in the context of EM, a disease in which the components of MF are hypothesized to play a key role in lesion establishment (Sampson, 1927). Despite the chronic inflammatory environment associated with EM, MF-sEVs from affected individuals exhibited comparable concentration and size distribution to those from healthy controls. However, molecular characterization revealed a significant reduction in CD63 expression and a downward trend in CD9, CD81, and Syntenin-1 levels, suggesting a shift in vesicle subpopulations. Given that CD63 and Syntenin-1 are closely linked to exosomal biogenesis (Mathieu *et al.*, 2019, 2021), these alterations may reflect disease-associated changes in endosomal trafficking or extracellular vesicle release mechanisms.

Transcriptomic profiling of sEVs cargo provided additional insights into the role of MF-derived sEVs in EM. The significant segregation of MF-EM-derived sEVs from healthy controls in PCA indicates a distinct disease-associated transcriptomic signature. Differential expression analysis revealed enrichment in biological processes related to cell motility, migration, and angiogenesis, aligning with key pathological features of EM (Saunders and Horne, 2021; Taylor *et al.*, 2021). This is supported by some upregulated mRNAs such as *TGFBI*, *VEGFA*, and *IL1B*, which have been shown to participate in EM-associated processes like fibrosis, angiogenesis, and inflammation, respectively (Zhang *et al.*, 2023). The over-enrichment of proangiogenic factors, both mRNAs and lncRNAs, suggests that MF-EM-derived sEVs may actively contribute to vascular remodeling within ectopic lesions of EM patients. Functional validation *in vitro* demonstrated that sEVs from EM patients significantly enhanced endothelial tubule formation, further supporting their proangiogenic potential. The identification of disease-associated molecular signatures in MF-sEVs highlights their potential as biomarkers for EM. Current diagnostic approaches rely on invasive laparoscopic procedures, often leading to significant diagnostic delays. The ability to non-invasively isolate and profile sEVs from MF offers a promising alternative for disease detection and monitoring. While the transcriptomic data provide valuable insights into the molecular landscape of MF-derived sEVs in EM, it is important to underscore that this component of the study represents a discovery-phase analysis. The cohort size used to obtain the transcriptomic data (n = 3 controls and n = 3 EM patients) limits the statistical power, and therefore, the identified molecular signatures should not be interpreted as definitive biomarkers. Instead, they offer a foundation for future research. Independent validation in larger, well-characterized cohorts will be essential to determine the reproducibility and clinical utility of these candidate markers. In this context, targeted validation of key upregulated genes, such as *VEGFA*, *TGFBI*, and *IL1B*, through RT-qPCR, would be a logical next step to advance the biomarker potential of MF-sEVs.

The enrichment of proangiogenic transcripts in MF-EM-derived sEVs suggests that their cargo may provide clinically relevant insights into disease progression and further supports the notion that sEVs reflect the functional and molecular state of their cells of origin carrying disease-specific cargo such as RNAs and proteins that influence recipient cells (Berumen Sanchez *et al.*, 2021; Clos-Sansalvador *et al.*, 2022). Although the precise target cells of MF-derived sEV in EM remain unidentified, it is plausible that their proangiogenic effect is mediated by their uptake into endothelial or stromal cells at ectopic sites. Integrins expressed on the surface of sEVs have been shown to mediate tropism and organ-specific uptake in other pathological contexts, such as cancer (Hoshino *et al.*, 2015), and may play a similar role in directing sEVs toward the vascular compartment in endometriotic lesions. Future studies focused on integrin profiling and uptake mechanisms may help clarify these targeting pathways. Further studies are needed to validate these findings in larger cohorts and to determine whether specific sEVs biomarkers can reliably distinguish EM from other gynecological conditions. Consistent with our findings, a recent study also successfully isolated and characterized MF-sEVs in the context of EM, with no significant differences observed in MF-sEVs concentration or particle size (Gurung *et al.*, 2025). While the present work identified transcriptomic changes on MF-sEVs from healthy and EM patients, Gurung and colleagues focused on their protein content, revealing an under-enrichment of proteins involved in immune response, programmed cell death regulation, cell polarity maintenance, and actin cytoskeleton organization. Moreover, *in vitro* experiments demonstrated that MF-sEVs from women with EM contribute to mesothelial barrier dysfunction, potentially increasing peritoneal adhesiveness and susceptibility to endometrial cell attachment. Despite their different approaches, both studies confirm that sEVs can be reliably isolated from MF and play a role in key biological processes relevant to EM, reinforcing the significance of MF-sEVs in disease pathogenesis. It is important to note that while our study included surgical/ultrasound validation (for controls and EM patients) and clinician-reported disease staging, Gurung *et al.* (2025) relied on self-reported diagnoses from both healthy individuals and women with EM. This distinction is important, as asymptomatic cases of EM have been reported (Rawson, 1991).

Despite the promising implications of this study, several limitations should be considered. The sample size, while sufficient for initial characterization, requires expansion to ensure broader applicability of the findings. This is particularly relevant for the RNA-seq component, which, given the small cohort size, must be interpreted as hypothesis-generating. While it offers valuable insights, the transcriptomic analysis lacks sufficient power for biomarker discovery and will require external validation. Additionally, while functional assays demonstrate the proangiogenic effects of MF-EM-derived sEVs, *in vivo* studies are necessary to confirm their role in lesion vascularization and disease progression. Lastly, while we identified alterations in sEVs cargo and function, future work should focus on elucidating the precise molecular mechanisms through which these vesicles mediate their effects on recipient cells. Another limitation of this study is the absence of data on medication use among participants. Analgesics, anti-inflammatory agents, or hormonal therapies could modulate both pain perception and potentially the composition of sEVs. Consequently, we cannot exclude the possibility that pharmacological treatment influenced either symptom persistence or the molecular profiles observed in MF-sEVs. Future studies should include detailed information on drug use and treatment response to clarify these potential confounding effects. Additionally, intracellular compartment markers were not assessed as part of MF-sEVs purity evaluation, which constitutes a minor limitation. However, these markers are considered optional under MISEV2023 guidelines, particularly for isolates derived from biofluids such as MF plasma (Welsh *et al.*, 2024).

In conclusion, this study establishes MF as a novel and abundant source of sEVs, providing an accessible, non-invasive means to investigate extracellular vesicles in gynecological health and disease. Our findings reveal that sEVs-enriched isolates from women with EM exhibit molecular and functional alterations, including enhanced proangiogenic potential, which may contribute to disease pathogenesis. Moreover, the unique transcriptomic signatures identified in MF-EM-derived sEVs highlight their potential as disease biomarkers. Future research aimed at validating these candidate signatures in larger cohorts and elucidating the mechanistic roles of sEVs in EM will be essential for translating these discoveries into diagnostic and therapeutic applications.

## Supplementary Material

hoag020_Supplementary_Data

## Data Availability

The data that support the findings of this study are openly available in NCBI Gene Expression Omnibus (GEO) at https://www.ncbi.nlm.nih.gov/geo/query/acc.cgi?acc=GSE310627, reference number GSE310627.
