# Correction to: Menstrual fluid-derived small extracellular vesicles: a novel reservoir with distinct molecular signatures and implications for endometriosis etiopathology

**DOI:** 10.1093/hropen/hoag091

**Published:** 2026-10-19

This is a correction to Vicente Peragallo-Papic, Paz Cerda-Castro, Aliosha I Figueroa-Valdés, Hugo E Tobar, Patricia Valdebenito, Manuel B Donoso, Benjamín Bustos, Javiera Yakcich, Reyna Peñailillo, Gino Nardocci, Stephanie Acuña-Gallardo, Sebastián E Illanes, Francisca Alcayaga-Miranda, Lara J Monteiro, Menstrual fluid-derived small extracellular vesicles: a novel reservoir with distinct molecular signatures and implications for endometriosis etiopathology, *Human Reproduction Open*, Volume 2026, Issue 2, hoag020, https://doi.org/10.1093/hropen/hoag020.

The RNA sequencing data deposited in the NCBI GEO repository under accession number GSE310627 and presented in the originally published version of Supplementary Table S1 were derived from menstrual fluid-derived mesenchymal stem cells instead of menstrual fluid-derived small extracellular vesicles.

This error resulted from the datasets being generated from the same patient cohort and stored in files with similar naming conventions. Consequently, the incorrect dataset was inadvertently uploaded during the data repository submission process and was then included in the submitted and subsequently published version of Supplementary Table S1.

Both NCBI GEO GSE310627 and Supplementary Table S1 have now been corrected to include the menstrual fluid-derived small extracellular vesicle RNA sequencing dataset.

This correction does not affect any results, analyses, or conclusions presented in the manuscript and does not alter the scientific message of the study in any way.

The authors apologize for any inconvenience this error may have caused.

## Supplementary Material

hoag091_Supplementary_Data

