# Supplementary figures and images for: Menstrual fluid-derived small extracellular vesicles: a novel reservoir with distinct molecular signatures and implications for endometriosis etiopathology
Source: Hum Reprod Open. 2026 Mar 15;2026(2):hoag020. doi: 10.1093/hropen/hoag020 (PMC13038252; doi:10.1093/hropen/hoag020)

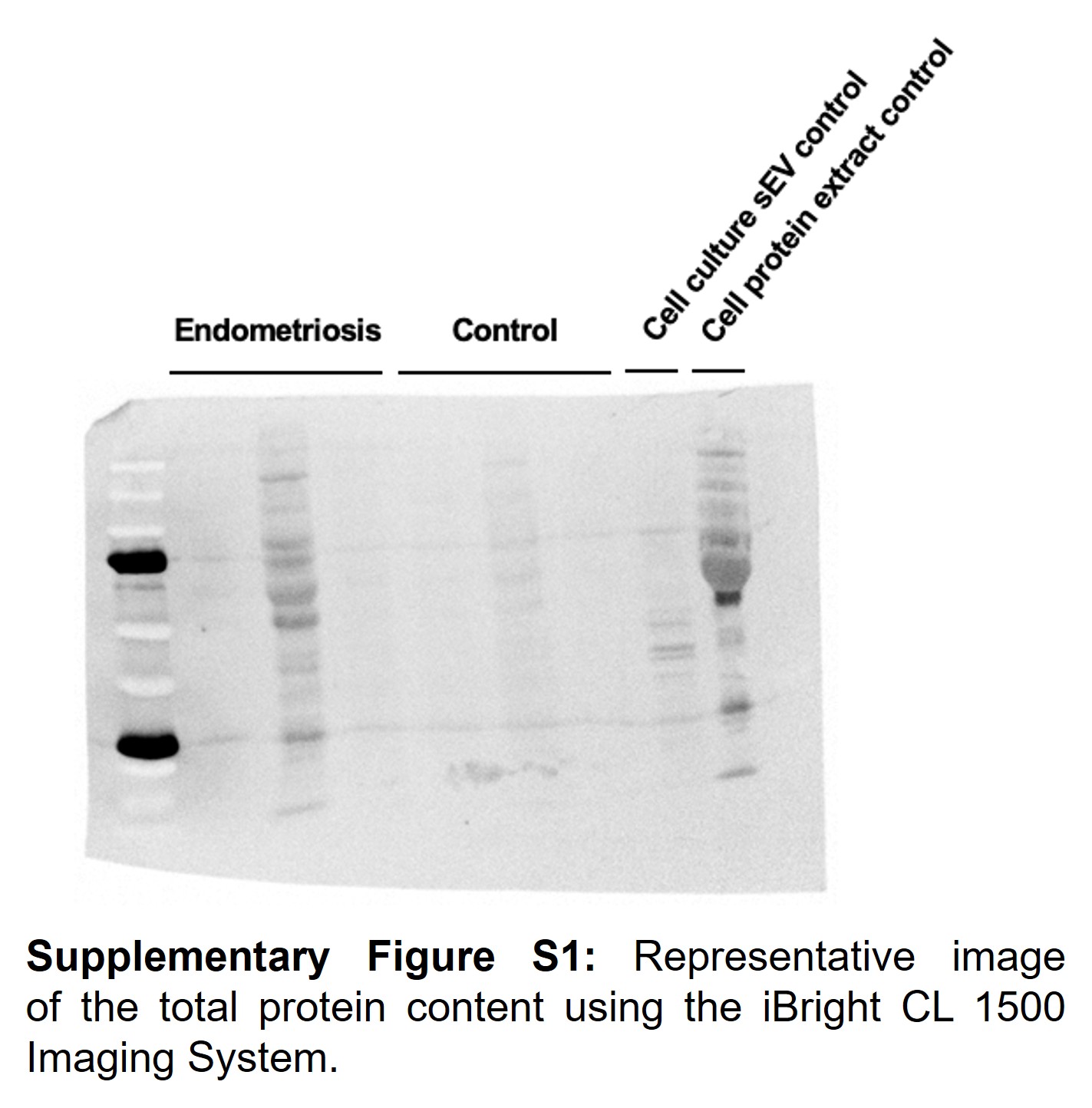

Supplement: hoag020_Supplementary_Data [file hoag020_supplementary_data.zip › Supp FigS1.jpg]

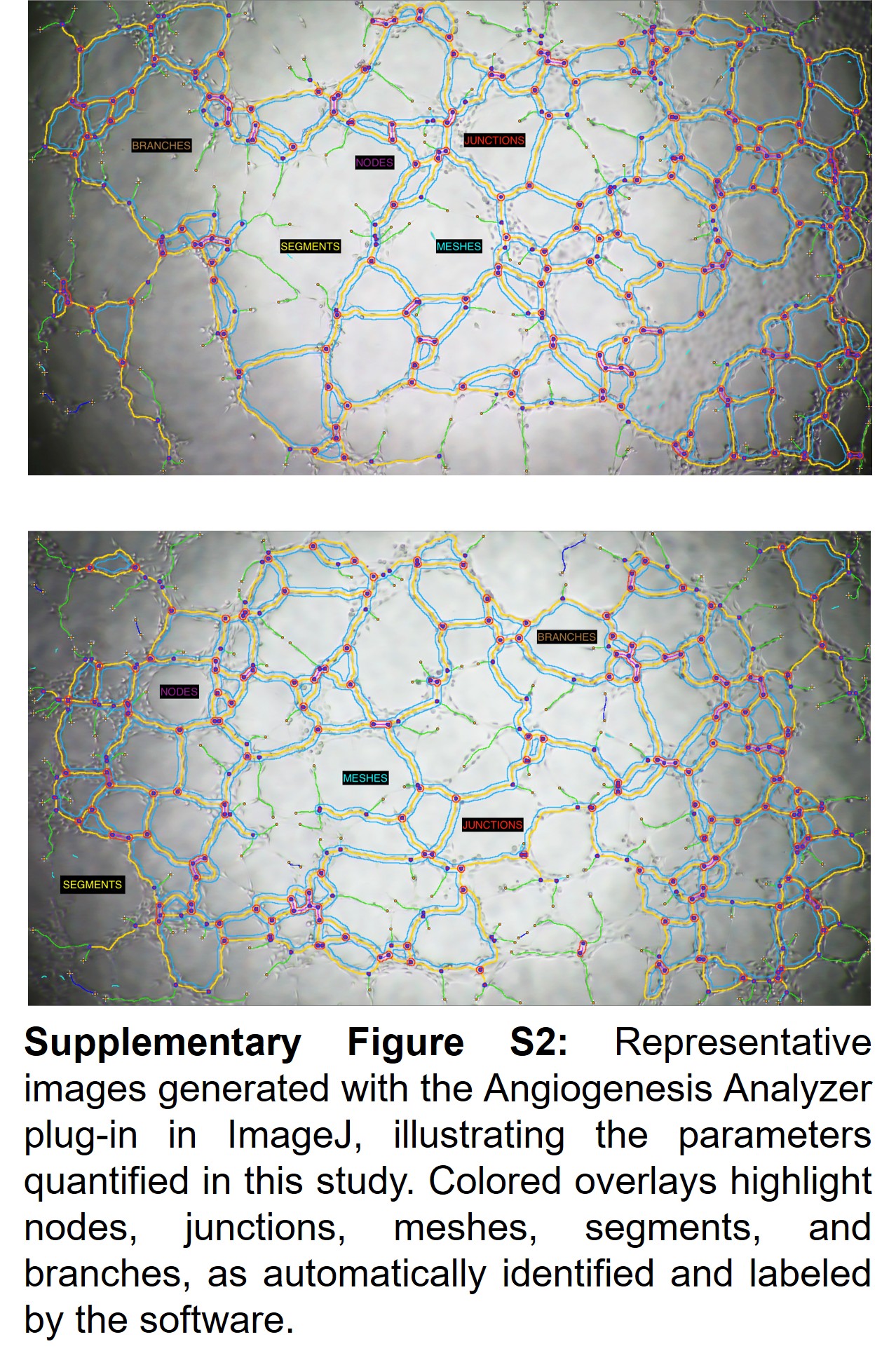

Supplement: hoag020_Supplementary_Data [file hoag020_supplementary_data.zip › Supp FigS2.jpg]

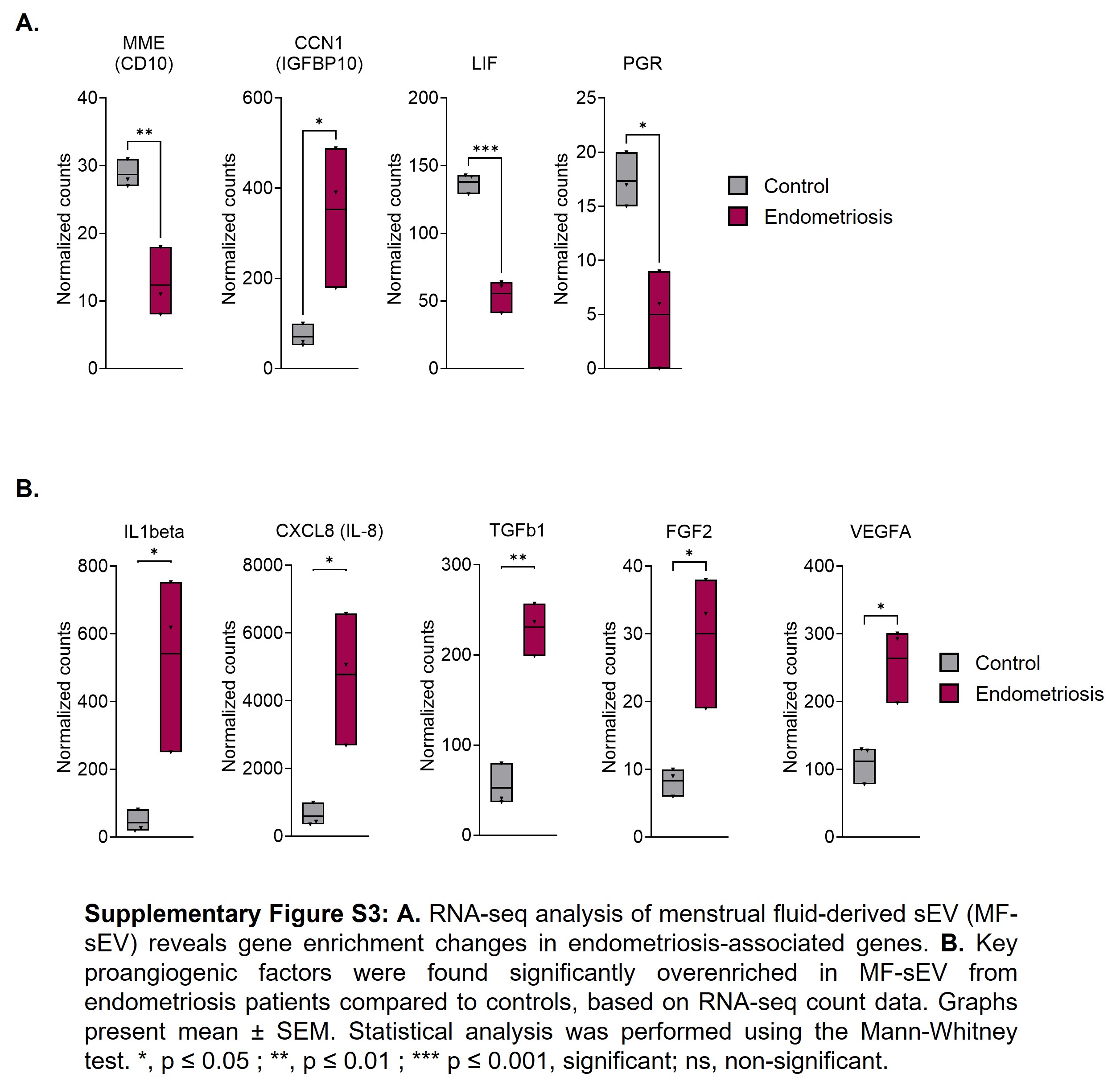

Supplement: hoag020_Supplementary_Data [file hoag020_supplementary_data.zip › Supp FigS3.jpg]
